# Direct reprogramming of induced neural progenitors: a new promising strategy for AD treatment

**DOI:** 10.1186/s40035-015-0028-y

**Published:** 2015-04-18

**Authors:** Siqiang Lai, Min Zhang, Dongsheng Xu, Yiying Zhang, Lisha Qiu, Changhai Tian, Jialin Charlie Zheng

**Affiliations:** Tenth People’s Hospital affiliated to Tongji University School of Medicine, Shanghai, 200072 China; University of Nebraska Medical Center, Omaha, NE 68198-5930 USA

**Keywords:** Alzheimer’s disease, Stem cell-based therapy, Induced neural progenitor cells

## Abstract

Alzheimer’s disease (AD) is a prominent form of dementia, characterized by aggregation of the amyloid β-peptide (Aβ) plaques and neurofibrillary tangles, loss of synapses and neurons, and degeneration of cognitive functions. Currently, although a variety of medications can relieve some of the symptoms, there is no cure for AD. Recent breakthroughs in the stem cell field provide promising strategies for AD treatment. Stem cells including embryonic stem cells (ESCs), neural stem cells (NSCs), mesenchymal stem cells (MSCs), and induced pluripotent stem cells (iPSCs) are potentials for AD treatment. However, the limitation of cell sources, safety issues, and ethical issues restrict their applications in AD. Recently, the direct reprogramming of induced neural progenitor cells (iNPCs) has shed light on the treatment of AD. In this review, we will discuss the latest progress, challenges, and potential applications of direct reprogramming in AD treatment.

## Introduction

Alzheimer disease (AD) is an aging-associated disorder with an incidence of 13% in people over 65 years of age [1]. In most countries, people with AD are a heavy burden to their families and the society. In China, the number of patients with AD and other dementias will reach an estimated 18 million by 2030 [2]. Thus, it is urgent to seek effective therapeutic strategies to cure this intractable disease. Although the neuropathogenesis of AD remains largely unknown, increasing evidence suggests that the accumulation and deposition of β-amyloid protein (Aβ), caspase activation, mitochondrial dysfunction, and neuronal loss contribute to the neuropathogenesis of AD. Specifically, the accumulation of Aβ in the brain is always believed to be the primary factor that triggers local inflammatory response and the extent of synaptic and forebrain cholinergic neuron loss [3-7], which cause direct decline in cognitive function. Currently, the chemical treatments of AD mainly include: (i) NMDA receptor channel blocker, such as Memantine [8,9] (antagonist to glutamate NMDA receptors). (ii) Enhancing the function of cholinergic neurons [10], such as Donepezil [11], Tacrine [12], Galanthamine [13], Rivastigmine [14], Huperzine A [15] (inhibitors of acetylcholinesterase, AChEI). (iii) Blocking Aβ’s production and decreasing its aggregation [16], such as Solanezumab [17] (humanized anti-Aβ monoclonal antibody), Bapineuzumab [18] (humanized anti-Aβ monoclonal antibody), Semagacestat [19] (small-molecule γ-secretase inhibitor). Unfortunately, these drugs have failed clinical trials, because they did not improve cognitive function. E.g., Semagacestat presented side effect, such as skin cancers and infections [17-19]. (iv) Scavenging free radical [20,21] such as N-acetyl-L-cysteine [22,23]. (v) Immune modulating [24], such as nonsteroidalanti-inflammatory drugs (NSAIDs) [25]. Although these treatments can alleviate symptoms to a certain extent (see Table 1) [26], they are incapable of preventing the degeneration of neurons and replacing the impaired ones in AD brains [27]. Stem-cell based therapy will provide a potential strategy for AD treatment, which is different from the chemical treatments.

**Table 1:** **Therapeutic effects of traditional treatments and stem cells-based therapies for AD**

**Therapeutic effects**	**Chemical treatments**	**Stem-cells based therapies**
Neuron replacement	None	ESCs
		NSCs
		MSCs
		iPSCs
		iNPCs
Aβ’s reduction	Solanezumab (clinical trials failed)	NSCs
	Bapineuzumab (clinical trials failed)	MSCs
	Semagacestat (clinical trials failed)	
Neuron protective/neurotrophic action	Memantine	MSCs
	Donepezl	
	Tacrine	
	Galanthamine	
	Rivastigmine	
	Huperzine A	
	N-acetyl-L-cysteine	
Immune modulating	Nonsteroidalantiinflammatory drugs	MSCs

### Current situation of stem cell-based therapies for AD

**Figure 1:**
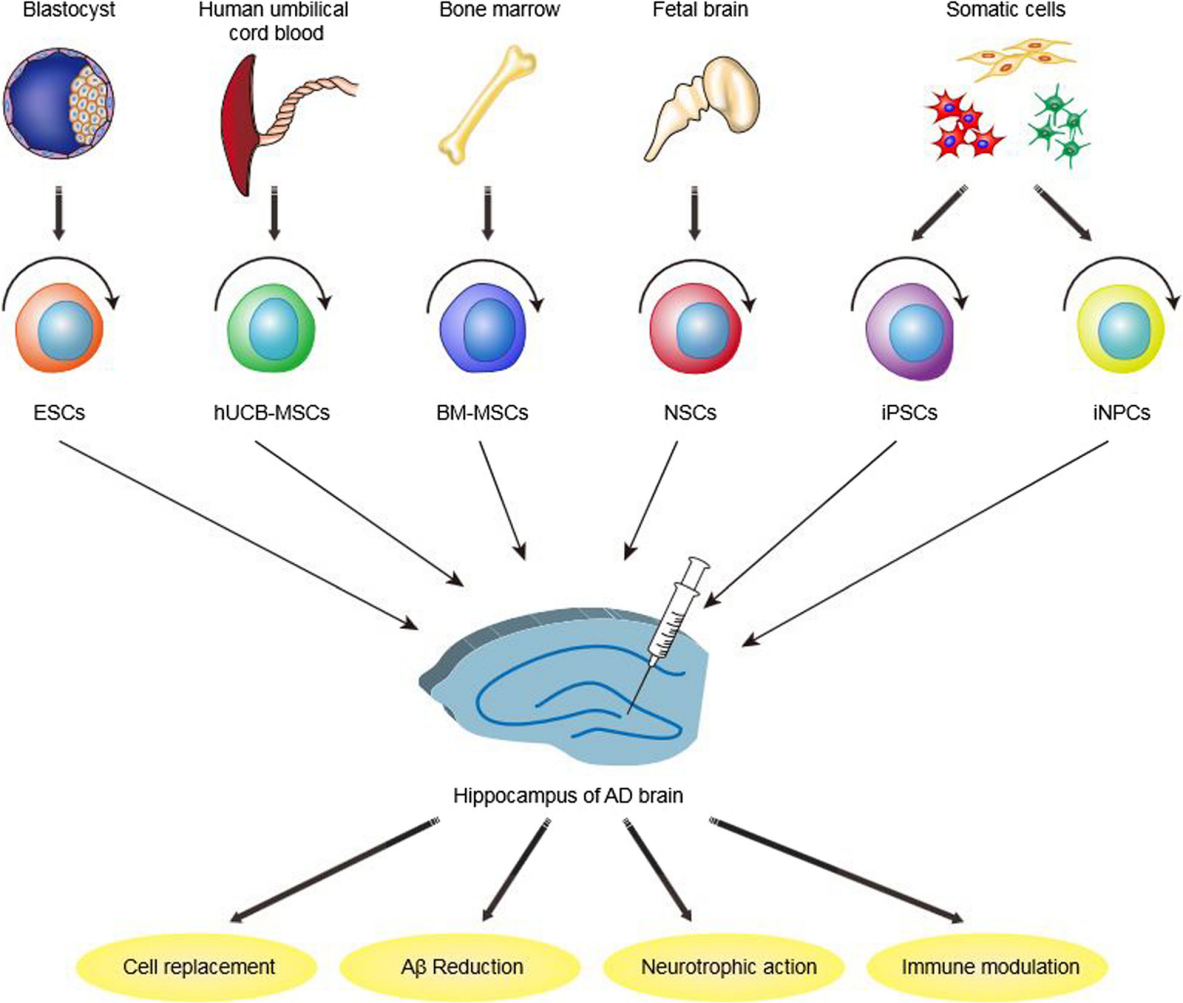
Current situation of stem cell-based therapies for AD. Stem cell-based therapies for AD can be achieved by cell replacement, Aβ reduction, neurotrophic action and immune modulation. ESCs, NSCs, MSCs, iPSCs, and iNPCs have the capacity to differentiate into cholinergic neurons to replace the apoptotic ones after transplanted. NSCs and MSCs are able to reduce Aβ or tau’s level. MSCs can play a positive role in neuroprotection and immune modulation.

Increasing evidence suggests that embryonic stem cells (ESCs), neural stem cells (NSCs), mesenchymal stem cells (MSCs), and induced pluripotent stem cells (iPSCs) have potential for AD treatment. These cells can improve the ability of spatial learning and memory for animals [28-37] by cell replacement [28,29], Aβ reduction [30-33,38], neurotrophic action [31] and immune modulation [34,39-41] (see Table 2) (Figure 1).

**Table 2:** **Stem cells-based therapies for AD**

**Stem cell types**	**Sources**	**Advantages for clinical treatment**	**Limitations**
ESCs	Blastocyst	Low immunogenicity	Ethical issues
		High capacity of pluripotency	Difficult to get enough cells
			Tumorigenicity
NSCs	Fetal brain	Low immunogenicity	Immune rejection
		Capacity of Aβ reduction	Ethical issues
		Low tumorigenicity	Difficult to get enough cells
MSCs	Bone marrow	Low immunogenicity	Low differentiated efficacy into neurons
	Human umbilical cord blood	No ethical issues	Injure patients to harvest BM-MSCs
		Capacity of Aβ reduction	Very limited source of hUCB-MSCs
		Immune modulation	
iPSCs	Somatic cells	No immunogenicity	Tumorigenicity
		No ethical issue	Low reprogramming efficacy
		High capacity of pluripotency	Low differentiation efficacy into specific neurons
iNPCs	Somatic cells	No immunogenicity	Low reprogramming efficacy
		No ethical issue	
		Abilities to differentiate into region- and subtypes-specific neurons	
		Direct reprogramming *in vivo* is simpler, quicker, safer, and harmless, as well as avoiding challenges of transplanted cells.

After transplanted, ESCs, NSCs and bone marrow derived-MSCs (BM-MSCs) can survive well and migrate to various brain regions [28], where they differentiate into cholinergic neurons, restore hippocampus synaptic density, and improve spatial learning and memory abilities for animals [28,29,33]. Moreover, NSCs and MSCs also reduce Aβ or tau pathology by phagocytic activity of astrocytes derived from transplanted NSCs [30-32] or microglia activation mediated by grafted MSCs to retard inflammatory processes [33,34,38-41]. Meanwhile, transplanted NSCs also secrete a series of neurotrophic factors, such as GDNF, BDNF and MANF [30-32], supporting the grafted cells to create more functional cholinergic neurons. Moreover, grafted human umbilical cord blood-derived MSCs (hUCB-MSCs) can also ameliorate the pathogenesis of AD by reducing the apoptosis and proinflammatory cytokines, increasing anti-inflammatory cytokines [39,40] and modulating oxidative stress [41]. Although the iPSCs technology has opened a new window for AD treatment, and newly generated neurons from iPSCs of familial AD patients also expressed MAP2 and β III-tubulin, formed functional synaptic contacts, and exhibited normal electrophysiological activity *in vitro*, these neurons showed similar cellular pathological feature with those in AD patients [42]. These studies suggest that iPSCs derived from AD patients may not be suitable for their own treatment.

Although ESCs, NSCs, MSCs, and iPSCs have some advantages in AD treatment, there are also problems that need to be solved before transplantation (also see Table 2). Currently, the ethical issues and immune rejection for ESCs and NSCs remain concerns, and also low differentiation efficiency for neurons due to lineage barriers and the limitation of cell source will be a challenge for MSCs [41]. Furthermore, the safety issue and low efficiency of iPSCs into subtype specific neurons will also limit its application in AD treatment.

### Generation of induced neural progenitors (iNPCs) by direct lineage conversion

**Figure 2:**
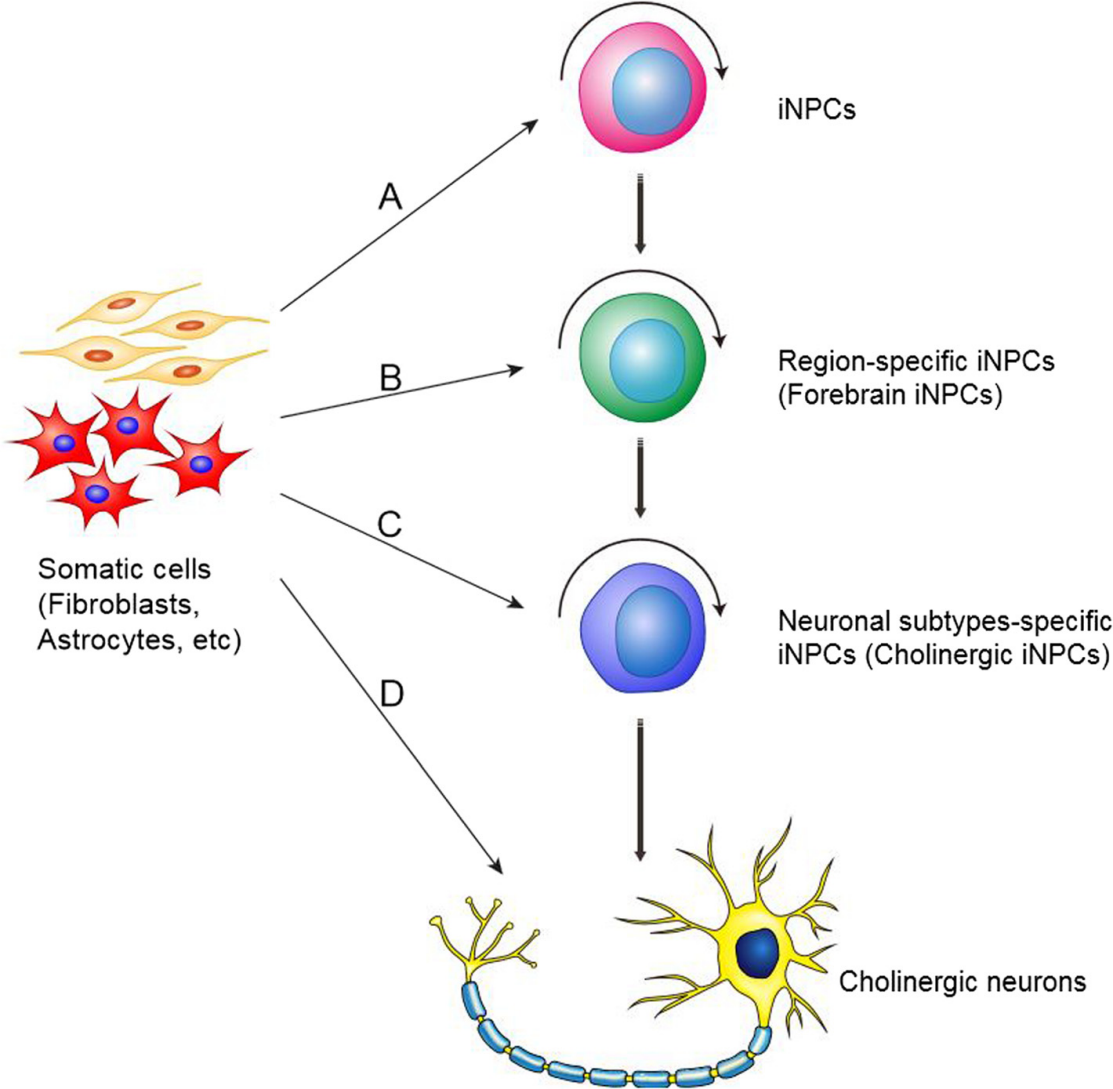
Strategies for direct reprogramming of iNPCs from somatic cells. iNPCs generated from different strategies. **(A)** Direct reprogramming of iNPCs by ectopic expression of defined transcription factors. **(B)** Direct reprogramming of region-specific iNPCs by expression of lineage-specific transcription factors. **(C)** Direct reprogramming of neuronal subtypes-specific iNPCs by using sets of defined transcription factors. **(D)** Generation of neuronal subtypes through direct reprogramming *in vitro* and *in vivo.*

Although functional neurons have been successfully generated through direct reprogramming [43], the low yield and non-proliferative nature of neurons derived from direct reprogramming limit its broad application in cell transplantation therapy of AD. Recently, progress suggests that induced neural progenitors (iNPCs) that give rise to all types of neural cells hold promising therapeutic effects on AD [44-46]. In our laboratory, we have been one of the first groups in the world to successfully convert somatic cells into iNPCs by ectopic expression of defined transcription factors, which share high similarities with primary neural progenitors in proliferation, self-renewal, and differentiation abilities [47,48]. Meanwhile, Pei’s lab successfully achieved iNPCs from mouse embryonic fibroblasts by chemical cocktails under a physiological hypoxic condition, without introducing expression of exogenous genes. These chemical-induced NPCs (ciNPCs) resembled mouse brain-derived NPCs in both cell properties and gene expression profiles [49]. These strategies avoid the ethical issue and reduce the risk of tumor formation [50,51]. Recently, we have been working on the direct reprogramming of somatic cells into region-specific iNPCs and subtype-specific iNPCs by ectopic expression of defined transcription factors. Hopefully, these iNPCs will have high differentiation efficiency for region-specific or subtype -specific neurons, and significantly improve the therapeutic effects in AD (Figure 2). Although multipotent neural stem/progenitor cells (NSCs/NPCs), including iNPCs that give rise to all types of neural cells hold promising therapeutic effects on AD, the specificity and efficiency induction of homogeneous cholinergic neurons generation from NPCs/iNPCs remain a challenge. Studies have showed that NSCs/NPCs respond poorly to pre-patterning morphogens with low efficiency for specific neuronal subtypes, and are prone to more glial-restricted states under typical culture conditions *in vitro* [52]. Moreover, grafted NSCs/NPCs are more likely to terminally differentiate into astrocytes rather than functional neurons in response to injury [53,54]. Therefore, stem cell-based therapies for AD based on the regeneration of specific neuronal subtypes, such as forebrain cholinergic neurons, will be more attractive. Although the major pathogenesis of AD was characterized by the selective degeneration of basal forebrain cholinergic neurons, recent study has demonstrated that selective degeneration of septal and hippocampal GABAergic neurons in a mouse model of amyloidosis and tauopathy has also been detected [55]. Thus, the direct conversion of GABAergic neural progenitor can be used an alternative strategy for AD treatment. Recently, neural conversion from somatic cells can also be successfully achieved *in vivo* [56-59], suggesting that it may be feasible to convert activated astrocytes into region- or subtype-specific iNPCs in the AD patients’ brains *in vivo.* These studies provide a simpler, quicker, and safer therapeutic strategy, which will allow us to directly inject defined factors in AD brain to switch the active astrogliosis into neurogenesis in the future, such as forebrain cholinergic neurons, avoiding cell transplantation.

In AD brain, the disease-related microenvironment, including aggregation of Aβ and inflammatory reaction, may decrease the proliferation and neurogenesis of transplanted cells, which will affect the treatment efficiency of AD. It is possible to improve the efficiency of iNPCs-based therapy by modulating the microenvironment via the use of a neurotrophic factor, Aβ-clear cells, and gene-engineered cells.

### Conclusion and prospective

Progresses in the stem cell field have opened new windows to generate region-specific and subtypes-specific neural progenitors through direct reprogramming from somatic cells, which will set up a new concept for AD treatment. Moreover, instead of cell transplantation, directly reprogramming activated astrocytes in the pathological site of AD brain into region- or subtype-specific iNPCs by the direct injection of defined factors *in vivo*, will be a promising strategy for AD treatment in the future. Furthermore, the therapeutic efficacy of stem cells can also be improved by modulating the disease-related microenvironment by improving the proliferation, differentiation, and self-renew of the transplanted cells. Although the transplanted iNPC will face pathological situation and many potential problems,the experience gained would set up a great foundation for our future in vivo reprogramming work. For further studies, we should try a more specific, more efficient and virus free delivering method for in vivo reprogramming. Taken together, the direct reprogramming of region-specific and neuronal subtype-specific neural progenitors *in vitro* and *in vivo* will be a potential strategy for the effective treatment of AD in the future.

## Abbreviations

AD: Alzheimer’s disease
Aβ: amyloid β-peptide, ESCs, embryonic stem cells
NSCs: Neural stem cells
MSCs: Mesenchymal stem cells
iPSCs: Induced pluripotent stem cells
iNPCs: Induced neural progenitor cells
BM-MSCs: Bone marrow-derived MSCs
hUCB-MSCs: Human umbilical cord blood-derived MSCs
